# Co-creating Digital Stories With UK-Based Stroke Survivors With the Aim of Synthesizing Collective Lessons From Individual Experiences of Interacting With Healthcare Professionals

**DOI:** 10.3389/fresc.2022.877442

**Published:** 2022-05-30

**Authors:** Joseph Hall, Thilo Kroll, Frederike van Wijck, Helena Bassil-Morozow

**Affiliations:** ^1^Department of Media and Journalism, Glasgow School for Business and Society, Glasgow Caledonian University, Glasgow, United Kingdom; ^2^UCD Centre for Education, Research and Innovation in Health Systems (UCD IRIS), School of Nursing, Midwifery and Health Systems, University College Dublin (UCD), Dublin, Ireland; ^3^Research Centre for Health, School for Health and Life Sciences, Glasgow Caledonian University, Glasgow, United Kingdom

**Keywords:** digital storytelling, behavior change, participatory design, stroke, healthcare professionals, co-creation

## Abstract

**Background:**

Stroke survivor narratives can provide valuable insight into experiences of healthcare and beyond. There is need to further understand collective lessons from stroke survivor narratives, yet prior studies utilizing digital storytelling tend to not synthesize lessons from individual experiences. This study aims to develop a novel method to co-create digital stories with stroke survivors that will aim to synthesize and portray important collective lessons from individual stroke survivors' experiences of interacting with healthcare professionals.

**Methods:**

This study follows-up a qualitative study conducted with 30 stroke survivors exploring factors that help or hinder survivors to positively reconfigure their identity post-stroke. Five co-creation workshops were conducted with a subset of UK-based stroke survivors from this previous study. Participants were invited to join through: online workshops, an online bulletin board, and as an advisor. A four-stage workshop framework was developed through the integration of UK Design Council's Double Diamond method, digital storytelling strategies and the Behavior Change Wheel (BCW) framework for developing behavioral change interventions.

**Findings:**

Six online workshop participants (three male, three female; aged 33–63; time since stroke 2–16 years) co-created digital stories that share six collective lessons aimed at increasing empathy and encouraging behavior change in healthcare professionals (HCPs) working with stroke survivors. Online bulletin board participants (*n* = 1) and advisors (*n* = 5) supported the co-creation process. Collective lessons identified were: (1) Stroke has a variety of symptoms that must all be considered; (2) Stroke can affect anyone of any age and not just the elderly; (3) Assumptions should not be made about a survivor's lifestyle or habits; (4) It is important to acknowledge the person behind the stroke and ensure that they are communicated with and listened to; (5) Stroke survivors can often feel unprepared for the reality of life after stroke; (6) Adapting to life after stroke is a long-term process requiring long-term support.

**Conclusion:**

Stroke survivor stories highlighted preconceptions, attitudes and behaviors embedded within healthcare that negatively impacted their experiences and recovery. The novel methodology employed in this study enabled these stories to be synthesized into collective lessons to bring about improvements in these behaviors in future.

## Introduction

Stroke is a global healthcare issue that may result in serious, disabling consequences for a significant proportion of survivors. Stroke remains the second-leading cause of death and the third leading cause of disability worldwide (1). Across the globe, stroke-related disability is on the rise, in 2019 stroke resulted in 143 million disability-adjusted life-years (DALYs), meaning DALYs due to stroke have increased 32% since 1990 (1). The continued rise in DALYs is now coupled with the increasing prevalence and incidence of stroke within people younger than 70 years old (1, 2). Furthermore, innovations in acute stroke care have resulted in improved survival rates, which, combined with aging populations and a growing list of important risk factors for stroke, mean that the stroke survivor population is growing, living longer but with more acquired disabilities (1, 3, 4). In turn, this is creating a global scenario where more individuals have to come to terms with the potential long-term impact of stroke-related impairments, activity limitation and participation restrictions on their individual and social lives, for which a considerable proportion require some form of rehabilitation. However, rehabilitation professionals' primary focus tends to be on the recovery of cognitive and physical functioning, while the myriad of life challenges survivors face following stroke are rarely addressed in rehabilitation practice (5, 6).

HCPs often play a key role in supporting stroke survivors and their families during the hyper-acute phase of potentially life-saving treatment, recovery and rehabilitation, as they can help to identify the specific needs and priorities of those affected by stroke (7, 8). However, this is not always guaranteed as a lack of collaboration between stroke survivors, caregivers and HCPs has been observed across the stroke recovery pathway (9). Furthermore, depending on the time post-stroke, the nature of subacute phase of stroke rehabilitation care one receives is often centered around medical, cognitive and mobility concerns (6, 10). The holistic impact of stroke is often not integrated into the care a survivor receives prior to discharge, as important aspects of recovery, such as psychological support, are often lacking (8, 11). For survivors, coming to terms with the sudden, and often drastic, change they experience following stroke can result in a long-term existential challenge (12). Yet, the impact of stroke on an individual's subjective and social lives often only becomes apparent following discharge from a primary healthcare setting (13, 14). Following discharge, survivors often experience a significant decline in support and a disjointed continuity of care (9, 15). It is during this period that stroke survivors and their carers can report how a lack of co-ordinated post-stroke care can leave them feeling unsupported (16, 17) and it is during this time period that feelings of abandonment arise (18–20).

In recent years, efforts have been made to better understand stroke survivors' experience of care and their transition from initial acute services to long-term support after stroke (8, 19). This has seen increased attempts to embed a person-centered focus to support stroke survivors in their initial and continued rehabilitation with the intention of improving stroke survivors' adjustment (21–25). However, the COVID-19 pandemic has exacerbated the pre-existing disparity between acute and post-stroke support, as an increase in the lack of provision of, or access to, post-stroke support has been reported (26–28); further highlighting the urgent need for new insights into how stroke survivors can be better supported by HCPs as they rebuild their lives and identities positively following stroke.

Narratives of illness can provide frameworks to help display a patient's holistic circumstances, encompassing not only their medical problem but the existential dilemmas that often accompany an individual's illness experience (29). As a result, stroke survivor narratives can provide powerful insight into experiences of recovery, helping to display the needs of survivors and where the provision of support and care can be improved (30–33). Providing stroke survivors with the tools to craft insightful stories can enhance the understanding of a variety of illness experiences amongst different groups, including HCPs, and ultimately instigate beneficial behavior change in regards to how these groups interact with, and support, stroke survivors.

One such approach that enables individuals to craft and share their personal stories is digital storytelling. Digital storytelling is a collaborative and participatory method where participants create short stories that reflect an individual's or group's experience through a mixture of still or moving images supported by text and/or audio (such as voice-overs, music or sounds). This approach shifts participants into the role of active co-producers of knowledge, as resultant digital stories are created and curated by the individuals themselves. Essential to this process is ensuring that creative control is given to the individuals who are at the center of these stories, to make sure that accounts are authentic and emanate directly from the lived experience of those who are sharing their stories (34). Thus, digital storytelling can provide marginalized individuals, or groups, with a voice to challenge stereotypes and societal narratives that surround them with the hope of influencing how they are perceived (35–37). Subsequently, digital storytelling has been used as a method to elicit empathy toward certain groups, as individuals have the space to share important life experiences that can be used to educate and inform (38–41). This has resulted in digital storytelling being adopted as a technique to educate HCPs (42–45). Digital stories have the capacity to provide insight into personal experiences of an illness or condition while also informing HCPs about patients' perspectives of experiencing healthcare (46). However, there has been a lack of academic research into the use of digital storytelling as a method for educating HCPs working with people after stroke.

Individual stroke survivor stories are appearing more frequently in the public domain, as stroke charities have increasingly used personal narratives on their websites and promotional material to help raise awareness about the reality of living with stroke (47, 48). Furthermore, websites such as the *Patient Voices Programme* (49) display a variety of patient digital stories with the intention of acting as an educational resource for HCPs to access, including stroke-related digital stories. However, the stories hosted on the *Patient Voices* website often exist in isolation without an attempt to convey collective lessons that may be gleaned from these various important insights.

The inherent phenomenological nature of digital storytelling can make it difficult, or undesirable, to both sharpen the focus of, or to rigorously synthesize, the experiences of seldom heard groups, as it may lead to the dilution of the individual voices presented. Yet, while providing a platform to said groups is important, a platform alone cannot always guarantee that valuable insights will catalyze change. Thus, sharpening the focus of co-created digital stories with the specified purpose of synthesizing collective lessons can help to support not only giving a platform to seldom heard groups, but to target and inform change. As a result, this study not only seeks to capture the experiences of individual stroke survivors but to synthesize their stories to learn collective lessons that can help HCPs better support the stroke survivors they work with. Therefore, this paper seeks to address the following aims:

1. To design a novel co-creation method that integrates a combination of: digital storytelling, a published design framework for co-producing new content; and a published framework for designing behavior change interventions.
2. To apply this novel co-creation method to identify core collective lessons for healthcare professionals working with people after stroke through the synthesis of stroke survivors' experiences of interacting with healthcare professionals.

## Methods

### Ethics

This research study was granted ethical approval by Glasgow Caledonian University's Glasgow School for Business and Society Research Ethics Committee and the University Ethics Committee on the 11/12/2020 (Ref no: GSBS EC 016).

### Study Design

A four-stage process was used to co-create digital stories with stroke survivors. The structure of the design process followed the UK Design Council's Double Diamond framework (50), integrating elements of Michie et al.'s Behavior Change Wheel (BCW) (51) and digital storytelling methods (34).

The Double Diamond method is a universal, human-centric design framework that has been flexibly applied to various academic and non-academic fields (52), providing a bounded structure to engaging in a co-production strategy, which places stakeholders at the center of the design process. The Double Diamond method (50) is comprised of four generic stages: *discover, define, develop* and *deliver* that help guide the design process from exploring the initial problem to designing, and creating, a solution. The Double Diamond method (50) was used to frame the co-creation process; breaking the design process into four stages, where an online workshop was held at each design stage. In total, we held an introductory session as well as five online workshops (two workshops focused on the *discover* stage). Throughout the design process, aspects of Joe Lambert's (34) digital storytelling methodology were implemented to help support participants translate their experiences into digital stories and to understand the various aspects that are involved in the creation of digital stories.

Furthermore, to help increase the anticipated future impact of the co-created digital stories on HCPs interactions with stroke survivors, the BCW (51) was integrated into the design process. The BCW was developed as a framework to provide a systematic process for developing behavior change intervention strategies, beginning with the initial behavioral analysis itself to the design and implementation of a specific intervention strategy (53). The BCW was designed as a framework for a wide range of disciplines, such as: policy makers, intervention designers, researchers, practitioners and any other party interested in “systematically applying theory and evidence to designing and evaluating behavior change interventions” (53) (p. 13). In regards to this study, the BCW was integrated into the four design stages of the Double Diamond approach. There are eight steps to the BCW, how these various steps were integrated into the co-creation process is detailed further in Table 1.

**Table 1:** Integration of BCW into Double Diamond design process.

**Double Diamond workshop stage**	Behavior change wheel steps (51)^*^	**Workshop procedures**
Completed prior to co-creation process through previous study	Step 1 “Defining the problem”	i) Increasing empathy in interactions between HCPs and stroke survivors was identified as key focus prior to co-creation process. ii) Digital stories chosen as desired Mode of Delivery for intervention.
Introduction	-	i) Explanation of study and co-creation process, participant Q&As.
Stage 1: Discover	Step 2 “Select target behavior” Step 3 “Specify the target behavior” Step 4 “Identify what needs to change”	i) Identify important lessons (behaviors) from participants' experiences, as the most important to share with HCPs. ii) Further specify target behavior in regards to important lessons. iii) Identify what needs to change in regards to HCPs's behavior that can help encourage positive interactions with, and support for, stroke survivors.
Stage 2: Define	Step 5 “Identify intervention functions” Step 7 “Identify behavior change techniques” Step 8 “Mode of delivery”	i) Design how identified important lessons will be communicated through the digital stories. ii) Education pre-determined as intervention function due to mode of delivery being digital stories. iii) Content of digital stories defined by participants will help outline behavior change techniques
Stage 3: Develop	*Co-creation of intervention function (digital story)*	i) Participants are provided with support and advice toward creating their own digital story. ii) Participants begin gathering material for their own digital story. Lead researcher edits digital story content for participants.
Stage 4: Deliver	*Finalize intervention function (digital story)* Step 7 “Identify behavior change techniques”	i) Digital stories are finalized and reviewed. ii) Collective lessons learned are finalized with input from participants, leading to potential modification of behavior change techniques.

* *BCW Step 6 is not addressed as policy will not be targeted.*

### Participants

Participants consisted of a subset of 30 UK-based stroke survivors who previously participated in a qualitative, grounded theory study. That study used 60–90 min long, individual, semi-structured interviews focused on life experiences after stroke. It involved 14 women, 16 men; aged 31–86; 1–25 years post-stroke. They were recruited *via* community support groups, care homes, and online social media adverts and posts. To take part in the previous study, the following inclusion and exclusion criteria applied; participants had to: be aged 18 years or above, be 1 year or over since survivor's initial stroke, live in the UK. Those who were unable to provide informed consent, or had a severe cognitive or communicative impairment restricting participants from portraying their narrative would not be able to partake. Stroke survivors with aphasia were supported to join the project through the use of the communicative tool Talking Mat (54), however none of the participants that took part required it. Every eligible participant was invited to provide consent to be recontacted for this follow-up study.

Those who agreed to participate in this follow-up study could choose, depending on their preference, between three forms of participation: online workshops (max. six participants), an online bulletin board or as an advisor. As the online workshops had a maximum number of participants, the other participation routes were offered to facilitate the participation of all consenting individuals from the previous study. The online workshop participants were selected through a maximum variation sample, this took into account participant demographics, i.e., age, gender, time since stroke, occupational status, living arrangements, geographic location, and their associated score on either the Scottish (55) or English (56) index of multiple deprivation. To join the study *via* the online workshops or online bulletin board, participants required a device (such as a computer, tablet or smart phone) capable of connecting to the internet to facilitate video calls or access websites. The six participants who contributed through the online workshops were directly involved in the co-creation of digital stories. Additional participants that joined through the online bulletin board and as advisors helped provide feedback on the discussions that took place within the online workshops. Input of additional participants was sought following the completion of each online workshop.

### Setting

Due to the COVID-19 pandemic, all participant contact took place remotely. All discussions took place online or over the phone. All online workshops were hosted over a password-protected videocall platform called Blackboard Collaborate Ultra^TM^. The online bulletin board was hosted on an encrypted Padlet page that only study participants could access. Finally, discussions with advisors took place over a variety of secure videocall platforms that participants were able to access (Blackboard Collaborate Ultra^TM^, Zoom^TM^, or Skype^TM^).

### Summary of Design Process

The following sections will describe the methods utilized in each stage of the co-creation process, and how the BCW steps are addressed within each stage of this process.

#### Stage 1: Discover

The *discover* stage focused on identifying the most important lessons recorded in the previous study and from the participants' own experiences from interacting with HCPs. This began with initial discussions to explore the participants experiences in-depth. This initial discussion mirrored elements of a *story circle*, a digital storytelling process where participants share their personal stories and experiences, usually in a more complete narrative, to help participants reflect, and begin to build, their complete story (34). However, there are inherent difficulties with applying this technique when working with stroke survivors as there is potential for acquired cognitive impairments to impede stroke survivors' capacity to recall their own story at length or to fully absorb and reflect on another person's story. Therefore, in order to make this process more compatible with the participants' needs, participants were invited to recall their experiences in regards to the key findings being discussed. This helped to make discussions more manageable and focused so that participants were not overwhelmed with information.

Following initial exploratory discussions to identify important lessons, the workshop was structured to ensure that BCW Steps 2, 3, and 4 were addressed. This involved working with the participants to translate emergent categories from initial discussions into specific target behaviors relating to HCPs interactions with stroke survivors that the participants believe are most important to change.

##### BCW Step 2

To prioritize the target behaviors that emerged from the participants' experiences, multiple criteria were considered. Firstly, the centrality of the target behavior to the experiences of the participants was assessed (51). Secondly, the extent to which changing the identified behaviors would have a positive effect on interactions between HCPs and stroke survivors was considered. Thirdly, how feasible it is to influence the target behaviors through the chosen mode of delivery (digital stories) was discussed. Finally, the capacity for the selected target behavior may have on other relevant, selected behaviors was also considered (51). Through meeting these criteria, the co-created digital stories would have better potential at influencing the chosen target behaviors.

##### BCW Step 3

Following the identification and selection of target behaviors, Step 3 of the BCW specified each of these behaviors in greater detail in terms of: who, when, where and with whom, the behaviors are relevant (51).

##### BCW Step 4

Finally, what exactly needs to change in order to effectively modify the target behaviors was discussed. The COM-B model, a theoretical model used to understand how three components (Capability, Opportunity and Motivation) can physically and psychologically impact one's ability to perform a behavior (B), was used to support the consideration of how these target behaviors could be addressed (51). Due to the educational purpose of this project, and the mode of delivery being predetermined as digital stories, HCPs' psychological capability was identified as the component that would maximize the possibility of behavior change. Psychological capability refers to our knowledge, psychological strength, skills or stamina to engage in any particular behavior (51). The workshop thus explored what needs to change in regards to HCPs' psychological capability to encourage behavior change in the target behaviors identified. The issues identified formed the main focus of the digital stories.

#### Stage 2: Define

The *define* stage focused on co-creating how to communicate the important lessons identified during the *discover* stage through digital stories. A plan for the digital stories was constructed to help explain the content and structure. Embedded into these discussions were prompts to get the participants to consider aspects of Lambert's seven elements of digital storytelling (34), such as: story perspective, emotional content that will engage the audience, how to personalize a story, and pacing. Integrating these elements successfully into digital stories can help shape the cohesion and effectiveness of the narrative shared. Following this, the participants co-designed a framework that would help shape the construction of their digital stories and potential dissemination routes for the digital stories were considered.

##### BCW Step 5 and Step 7

As the co-created digital stories aim to change the psychological capability of HCPs, the most appropriate intervention function suitable in this format is education (51). Furthermore, as the intervention function will be education, the behavior change techniques (BCTs) selected will be related to this intervention function, dependent on the specific issues participants decided to highlight. Following the completion of Stage 1 and 2, the lead researcher (JH) began coding BCTs that were evident in the co-creation process. The BCTs were finalized following the completion of participant input during Stage 4. More information on this process can be found later in the Results section. BCW step 6 was not applicable to this study due to the study not aiming to design policy.

#### Stage 3: Develop

The *develop* stage focused on supporting participants in the creation of the digital stories. Topics covered in this workshop were: planning a story, writing a script, storyboarding, capturing audio, gathering or creating visual material, and ethical and copyright issues. With reference to Lambert's work on digital storytelling (34), exercises and discussions were held around these topics to help participants become familiar with the process of designing a digital story. A participant guidebook, purposefully written for this study and provided in a written and audio format, expanded on these topics and would help support participants in creating and gathering material for their digital story contribution outside of the workshop.

Following the conclusion of the online workshop, participants would then have a period of time to plan their digital story contribution and gather material they would like to include within it (audio, personal images, copyright free images etc.). The lead researcher was available throughout this period to discuss their ideas and provide any support in helping produce or gather material for their story. At the end of this period, the participants' digital story plan and material were given to the researcher who then edited these elements together. This decision was made as editing requires a higher degree of computer literacy and may be a difficult task depending on the nature of an individual's acquired disabilities or the capability of the technology they own. Once the editing process began, the researcher was in frequent communication with the participants to ensure the digital story met each participant's expectation.

#### Stage 4: Deliver

The *deliver* stage online workshop was focused around finalizing the digital stories, the synthesized collective lessons, and how they would be presented through the project website. At this stage, each online workshop participant was asked to view the other participants' completed digital stories and to consider whether any of the lessons needed to be changed or whether any new lessons needed to be added. This discussion was then the primary focus of the Stage 4 workshop.

To assist finalizing the synthesis of the collective lessons, the lead researcher (JH) conducted a thematic analysis of the digital stories to help verify existing lessons and to potentially uncover new common themes that exist between the participants' stories. This analysis was conducted using Braun and Clarke's (57) six-step framework (see Table 2), a recursive method in which subsequent steps may encourage the return to previous steps for further investigation. Any new themes noticed by the researcher would be raised with the participants during the workshop for their consideration and input.

**Table 2:** Braun and Clarke's six-step framework for conducting thematic analysis (57).

**Framework step**	**Step description**
Step 1: Become familiar with the data	Repeated and active reading through the data, prior to the development of codes.
Step 2: Generate initial codes	Coding features across dataset, collate data relevant to each potential theme.
Step 3: Search for themes	Collate codes into potential themes, gather all relevant data for these themes.
Step 4: Review themes	Check suitability of themes in relation to both coded extracts and across dataset, helping to create a thematic map of analysis.
Step 5: Define themes	Continued analysis aiming to refine each theme and overall analysis, resulting in clear definitions and names for themes.
Step 6: Write-up	Final analysis relating to wider research issue and literature. Selection of compelling quotes.

After the workshop, the lead researcher made any agreed upon adjustments to the digital story content and the description of the synthesized lessons learned. The participants' digital stories, and information on the synthesized lessons learned were then uploaded onto an encrypted project website which will be used as a dissemination source. This website includes both text and audio content (alongside the digital stories). Participants were then asked to view the website and provide feedback. Once any additional feedback was integrated, the website was finalized as a resource to be included within the intervention strategy.

##### BCW Step 7

Following the finalizing of the participants' stories and the synthesized collective lessons to be shared, the lead researcher coded the BCTs.

### Management of Co-creation Data

The co-creation process was captured *via* recordings of all relevant participant discussions with the online workshop, online bulletin board and advisors. This includes recordings of videocalls (including webcam footage) and phone calls were transcribed verbatim, as well as text transcripts from any relevant emails and contribution through the online bulletin board. To increase the credibility (58) of the data, detailed interview guides were used for each workshop. Following the completion of each workshop, a summary of the data collected was sent back to each participant for verification. To help the dependability and confirmability (58) of the findings, the transcripts were prepared and analyzed as quick as possible to best reflect discussions. Research notes were kept in regards to the procedure, process, and any observations by the researcher.

## Results

The purpose of this section is to highlight the participants' contribution throughout the co-creation process and to detail the final lessons the participants chose to highlight as the most important to share with HCPs working with people after stroke through the co-created digital stories.

### Participant Information and Workshop Attendance

Of the 29 participants from the previous study who provided consent to be contacted about this follow-up study, six provided informed consent to participate in the online workshops. These participants were composed of: three women and three men, aged 33–63, and time since stroke ranged between two to 16 years. Further information on the online workshop participants can be found in Table 3. Additional participants provided informed consent to participate through the online bulletin board (*n* = 1) and as advisors (*n* = 5). Seventeen participants contacted from the previous study were either unable to partake (*n* = 5) or did not respond (*n* = 12).

**Table 3:** Online workshop participant information.

**Participant**	**Age**	**Gender**	**Time since**	**Self-perceived**
**pseudonym**			**stroke**	**stroke severity**
Sandra	55	Female	15 years	Moderate
Alan	59	Male	2 years	Severe
Emma	33	Female	4 years	Severe
William	63	Male	3 years	Moderate
Jessica	33	Female	7 years	Severe
Ian	53	Male	1 year	Mild

Between June and November 2021, six online workshops took place. All six online workshop participants took part in the five initial online workshops that were held. However, one participant left the project due to medical reasons and five participants completed a digital story and partook in the final *deliver* stage workshop. Input of additional participants was sought following the completion of each online workshop.

### Co-creation Process

The following section will detail the decisions and input collected from the participants at each stage throughout the co-creation process that culminated in the final focus and content of the co-created digital stories.

#### Stage 1: Discover

During Stage 1 of the study, participants identified healthcare target behaviors that would help to increase empathy and encourage behavior change amongst HCPs working with people after stroke. During initial discussions, three themes were identified by the participants as the most important: experiences of misdiagnosis, communication between HCPs and stroke survivors, and feeling unprepared for life following discharge. To help achieve BCW steps 2 and 4, these key themes were translated into relevant target behaviors and what exactly needs to change to effectively modify the target behaviors was identified (see Table 4).

**Table 4:** *Discover* stage: what healthcare behaviors need to be changed when interacting with stroke survivors and how to influence change (BCW step 2 and 4).

**Identified healthcare target behaviors (BCW step 2)**	**COM-B components**	**What needs to change? (BCW step 4)**
1) HCPs making assumptions about stroke	Psychological capability	i) Raise awareness that stroke onset has a variety of potential symptoms. ii) Raise awareness that stroke can impact anyone of any age and not just the elderly. iii) Raise awareness that stroke is not always caused by someone's lifestyle or habits.
2) Managing conversations about post-stroke recovery	Psychological capability	i) Raise awareness about the impact conversations about post-stroke recovery can have on stroke survivors (i.e., feeling unprepared for life after stroke or on one's hopes for recovery)
3) Adequately preparing stroke survivors for discharge and life after stroke	Psychological capability	i) Raise awareness of how unprepared many stroke survivors feel when they return home following discharge from a primary healthcare setting. ii) Raise awareness of the importance of the psychological, emotion and social impact of stroke.

How the participants further specified the identified target behaviors (BCW step 3) can be found in Table 5. It is important to note that, while the identified target behaviors can be specified in particular circumstances, the participants identified that it is important to acknowledge the generic nature of these behaviors. This is because the target behaviors that have been identified deal with the interaction and general communication between HCPs and stroke survivors. As a result, these target behaviors have the capacity to be relevant at various points during interactions between HCPs and stroke survivors.

**Table 5:** Specifying target behaviors (BCW step 3).

**What target behavior?**	**Who**	**When**	**Where**	**With whom**
1) Challenge HCPs' assumptions about stroke	First responders and Healthcare staff interacting with stroke survivors	Always	Attending emergency call + primary healthcare setting	(Potential) stroke survivors and their families
2) Managing conversations about post-stroke recovery	Healthcare staff interacting with stroke survivors	Discussing recovery expectations	Primary and community healthcare setting	Stroke survivors and their families
3) The need to prepare stroke survivors for discharge and life after stroke	Healthcare staff interacting with stroke survivors	Preparing survivors for discharge from primary healthcare setting	Primary and community healthcare setting	Stroke survivors and their families

#### Stage 2: Define

**Figure 1:**
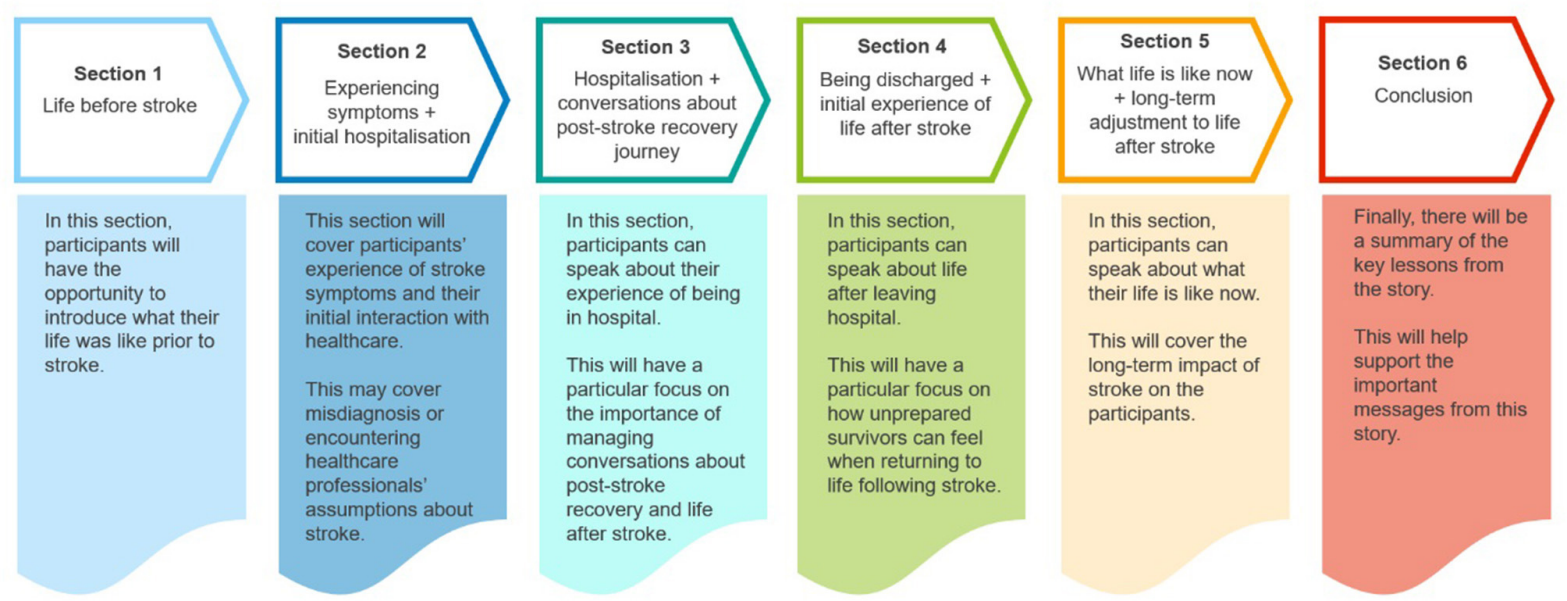
Co-created framework for digital stories.

During Stage 2, the participants developed a plan which aimed to address the issues outlined during Stage 1 through a linear story structure (Figure 1). This structure was decided upon as it would help to convey the various important issues identified by the participants within a coherent narrative of their experience from life before stroke to what life is like now. The target behaviors can be plotted onto various points of the chronological journey of their experience of life after stroke and are therefore integrated into the section design. This digital story plan would help participants to structure their own individual stories.

Furthermore, potential dissemination routes were discussed with participants. It was confirmed that a project website would host the participants' stories with information pertaining to the synthesized collective lessons. Links to the website would be shared on social media. Finally, a future online event would be hosted inviting HCPs to attend and provide input and feedback on the content of the digital stories and their collective lessons.

#### Stage 3 and 4: Develop and Deliver

Stage 3 focused around supporting participants to create their individual digital stories, the contents of which are discussed later. Following the completion of Stage 3, participants were asked to review the other participants' digital stories to consider if there were any new lessons that could be learned or amendments to previous lessons could be made. To support this discussion, the lead researcher conducted a thematic analysis (57) of the digital stories which resulted in three areas for further consideration being noted:

1) Feeling heard, acknowledged and listened to by HCPs was a significant theme across the digital stories.
2) Raising awareness around the importance of the psychological, emotional and social impact of stroke seemed to be part of a wider concern in regards to the ongoing, long-term impact of stroke.
3) While the participants identified raising awareness that stroke is not always caused by someone's lifestyle or habits as a key issue, none of the participants directly addressed this in their stories. However, it was noted that all five participants mentioned at the start of their stories that they lived a healthy lifestyle pre-stroke and it was identified as an important lesson to communicate during the co-creation process.

At the beginning of the Stage 4 workshop, participants were asked to discuss what additional themes they had noted. The participants also identified the importance of patient-centered communication. It was decided that this issue should be its own key lesson and that the previous lesson in regards to conversations about post-stroke recovery would be integrated into the lesson regarding how unprepared stroke survivors feel when they return home. Following this discussion, the lead researcher raised the importance of the long-term impact of stroke and then the issue of lifestyle and habits. The participants agreed that the initial identified area of raising awareness around the psychological, emotional and social impact of stroke could be seen as part of a wider issue in relation to the long-term nature of adapting to life after stroke. Participants then discussed that this issue is more pertinent for HCPs and that awareness about the need for long-term support following discharge should be raised. Finally, the issue noted in regards to raising awareness about stroke not always being caused by someone's lifestyle or habit was discussed. The conclusions of this discussion, and the other identified collective lessons, are covered in greater detail within the “Digital Story Analysis” section.

Following the finalizing of the participants stories and the collective lessons to be shared, the lead researcher coded the BCTs to satisfy Step 7 of the BCW. Three BCTs from the behavior change technique taxonomy v1 were identified (59). Table 6 shows the BCTs selected and their specific operationalisation. The digital stories and the collective synthesized lessons will help deliver these specific BCTs.

**Table 6:** Selected BCTs and operationalisation in designed intervention (BCW Step 7).

**Selected BCTs**	**Operationalisation of BCT**
Information about health consequences	i) Inform HCPs about the impact of failing to consider stroke symptoms other than “FAST.” ii) Inform HCPs that stroke can impact anyone of any age and not just the elderly. iii) Inform HCPs about the impact on stroke survivors of assuming stroke was caused by someone's lifestyle or habits. iv) Inform HCPs about the need to manage the transition following discharge and provide ongoing support.
Information about social and environmental consequences	i) Inform HCPs of the impact on stroke survivors' psychological and emotional wellbeing of hospitalization and experiencing the hospital environment. ii) Inform HCPs of how unprepared many stroke survivors are for the impact of stroke on their social lives and interactions.
Information about emotional consequences	i) Inform HCPs about the impact conversations about post-stroke recovery can have on stroke survivors' recovery goals and preparation for life after stroke. ii) Inform HCPs about the importance of creating positive personal relationships with stroke survivors. iii) Inform HCPs about the impact of actively listening to stroke survivors' input and acknowledging their subjective concerns. iv) Inform HCPs about the impact of stroke survivors feeling unprepared for life following discharge. v) Inform HCPs about the continued emotional impact of dealing with long-term changes post-stroke.

### Digital Story Analysis

A total of five individual digital stories were created during this process, lasting between 3 min 30 s and 6 min 4 s. Each digital story followed, or integrated, the structure that was designed by the participants to detail the collective issues that were highlighted over the course of the workshops. By the end of the co-creation process, the content of these stories had been synthesized into the six most important lessons that the participants identified as the most significant to be shared with HCPs (see Table 7). The purpose of this section is to detail the important lessons identified by the participants with the support of direct quotations taken from the participants' digital stories. Pseudonyms will be used where participants are referred to directly.

**Table 7:** Final six collective lessons chosen by participants to be addressed by digital stories.

**6 key collective lessons from digital stories**
i) Stroke has a variety of symptoms that must all be considered ii) Stroke can affect any of any age and not just the elderly. iii) Assumptions should not be made about a survivor's lifestyle or habits.
iv) It is important to acknowledge the person behind the stroke and ensure that they are communicated with and listened to.
v) Stroke survivors can often feel unprepared for the reality of life after stroke. vi) Adapting to life after stroke is a long-term process requiring long-term support.

#### Lesson 1: Stroke Has a Variety of Symptoms That Must Be Considered

The participants reported being misdiagnosed, often due to their varied symptoms being overlooked. As a result, they often did not receive the urgent medical assistance required. Misdiagnosis from HCPs was often found to result in a less serious diagnosis, such as a migraine. Subsequently, this could falsely reassure and discourage participants from seeking the urgent medical assistance their situation required; an experience William details in his story:

The diagnosis I'd been given by a variety of specialists was I was suffering from a migraine. I put any confusion, dodgy eyesight, and lack of articulacy down to my change in lifestyle. I hadn't even considered a stroke. (William, aged 63)

The false reassurance participants reported due to misdiagnosis furthered the delay in receiving urgent care. However, other participants were aware of the serious medical situation they were in, yet, participants described having to “fight for an ambulance” and “beg” reluctant paramedics to take them to hospital. These experiences highlight the potential for HCPs to not engage seriously with the concerns of potential stroke survivors, even when they are aware of the serious nature of their condition.

Participants believe their experiences of misdiagnosis were associated with HCPs not acknowledging their variety of symptoms as potential stroke symptoms. Many expressed this may be due to the association between stroke and the FAST (60) (Face, Arms, Speech, Time) mnemonic used to help HCPs quickly detect potential acute strokes through common symptoms that pertain to one's Face, Arms and Speech. The participants acknowledged the benefit of the FAST mnemonic, and wider health promotion campaign around it, in raising awareness of important stroke symptoms. The benefits of which are evidenced in Alan's experience as his family identified stroke and called an ambulance; while attending paramedics were quick to provide appropriate care. However, the participants identified the importance of HCPs acknowledging symptoms that extend beyond a person's Face, Arms and Speech. Participants believe not having typical FAST symptoms may have contributed to their initial misdiagnosis; an experience Jessica reflects in her story:

I would like people in the healthcare profession to…be aware of not just the FAST symptoms. I would like them to look at other symptoms, such as balance, vertigo and nausea. I might have told a different story if my stroke was recognized a lot sooner. (Jessica, aged 33)

As a result, acknowledging a variety of potential stroke symptoms, such as balance and vision, may be important to ensure those experiencing stroke receive vital care as soon as possible. Assumptions should not be made about the symptoms an individual is experiencing beyond FAST, and all potential symptoms that may indicate a stroke should be taken into consideration for diagnostic purposes. This is particularly important when first responders are interacting with people who may have had a potential stroke, as it may impact the speed at which critical care and treatment is received. Furthermore, it is important to acknowledge stroke misdiagnosis is often multifaceted, interwoven with potentially erroneous assumptions that can be made due to a stroke survivor's age or lifestyle (see Lesson 2 and Lesson 3 for more information).

#### Lesson 2: Stroke Can Impact Anyone of Any Age and Not Just the Elderly

Younger participants encountered stigma associated with the assumption that stroke is a disease of the elderly. Survivors expressed their belief that they were misdiagnosed as a result of their age, and not just due to the nature of their symptoms. Initially, paramedics were reluctant to take Jessica to hospital, she was 27 at the time of her stroke, recently becoming a mother a few months prior.

The paramedics came and took a look at the baby and said I was exhausted. They said to rest and get my doctor to come and visit me that afternoon. I begged to go to hospital and had my blood pressure done and that was fine and apparently I was FAST negative. I believe because of the age stigma associated to stroke that was my first let down. (Jessica, aged 33)

Jessica's experience continued after she arrived in hospital, as healthcare staff continued to misdiagnose her with a suspected ear infection and, as she deteriorated, meningitis. Jessica believes assumptions about her symptoms were made due to her age, delaying accurate diagnosis and the delivery of appropriate care. Similar experiences were reflected in Emma's story, as she, aged 29 at the time of her stroke, exhibited typical FAST symptoms but was initially diagnosed with a hemiplegic migraine and discharged, only for her symptoms to return and have the attendance of an ambulance denied. Their experiences show how age associated stigma can fuel assumptions about stroke's symptoms, subsequently amplifying the possibility of misdiagnosis.

However, stroke survivors can encounter age-related stigma throughout their interactions with HCPs, as Emma details:

One thing that was said to me a lot during my stay in hospital was that I was young to have a stroke, and this needs to change. (Emma, aged 33)

Emma reflects how unhelpful younger stroke survivors can find it whenever they are told that they are young compared to the average stroke survivor. This has the potential to ostracize younger patients and make them feel more alone in their experience, potentially making it harder for them to come to terms with having a stroke (61). This issue can also be compounded as stroke survivors experience care that is built for a population that is expected to be elderly.

Participants want stroke to no longer be treated as a phenomenon associated with the elderly; it should be regarded as a health issue that can impact anyone of any age. First responders should not make assumptions about a person's symptoms due to their age; this could lead to misdiagnosis and a delay in delivering vital treatment. Furthermore, once in a healthcare setting, survivors should be reassured and not made to feel like an outlier.

#### Lesson 3: Assumptions Should Not Be Made About a Survivor's Lifestyle or Habits

Each of the five participants expressed in their stories that they lived a healthy lifestyle prior to stroke; for example, William states “I'd always run and cycled…I'd always been fit” or as Emma explains that she spent her “free time being as active as possible” and had “a gym membership I took full advantage of.” While the participants did not specifically mention how this directly impacted their interactions with HCPs, it is important to acknowledge why the participants chose to include this information and why it was selected as an important lesson by the participants.

When prompted by the lead researcher, the participants explained that mentioning the healthy aspects of their lives prior to stroke was because they wanted to distance themselves from the possible assumption that their stroke will have been caused by lifestyle choices and engaging in behavior that could have contributed to having a stroke. This helps the participants to defend themselves from negative social connotations surrounding the cause of stroke and how blame can be placed on the individual who has experienced the stroke. As a result, the participants emphasized the healthy aspects of their lifestyle prior to stroke in order to disassociate themselves with the tainted and discounted labels attached to those stigmatized by society (62); which in this case, is stroke survivors who are deemed to be responsible for the cause of their stroke due to engaging in seemingly reckless and dangerous behavior. Furthermore, while lifestyle choices, such as: smoking, drinking, poor diet and lack of exercise, increase the likelihood of stroke and account for a significant percentage of strokes (63), there are many potential causes of stroke that should be acknowledged and considered.

In relation to HCPs, it is important that assumptions are not made about an individual's lifestyle, and how it may have caused their stroke; this can result in individuals believing that the stroke was their fault, even when they have not engaged in behaviors that are a risk factor for stroke. HCPs should be aware of the presence of potential stigma associated with stroke and its perceived cause, and how this may impact individual stroke survivors. Ultimately, if the cause of a stroke cannot be immediately identified, assumptions should not be made about the possible causes of a person's stroke. Where possible, HCPs should avoid situations where the individual can feel stigmatized and made to feel responsible for their stroke.

#### Lesson 4: It Is Important to Acknowledge the Person Behind the Stroke and Ensure That They Are Communicated With and Listened to

Stroke survivors expressed the benefits of interacting with HCPs who listened to their input and concerns. Previous lessons showed the significance of listening to stroke survivors during the initial onset of stroke to help deliver urgent care as soon as possible. However, ensuring survivors are listened to extends throughout all interactions with HCPs following stroke. Following initial hospitalization, Alan was unable to walk, talk, eat, and was partially paralyzed. However, he was conscious and was aware of everything going on around him:

I could hear and understand everything, but the medical staff didn't quite believe this, as they would have many conversations which I could hear but not comment on. Which was extremely frustrating and frightening. It was like being locked-in. (Alan, aged 59)

The lack of understanding Alan experienced fuelled concern he had in regards to the severity of the impact of stroke. However, Alan's relationship with the HCPs who were treating him changed after he was transferred to a new hospital.

The staff were keen to find out who I was, and from then on, I was treated like Alan, not a stroke patient. With the dedication of the staff, and the confidence they gave me to improve, I was taught to sit-up by myself, walk, talk, and have a whole new outlook post-stroke. (Alan, aged 59)

Initially, the healthcare staff's lack of understanding of Alan's capacity exacerbated the isolation he was experiencing following stroke. However, after transferring to another hospital, where he felt listened to and treated as an individual, he felt encouraged and supported by the staff. Experiences such as this show the impact positive, person-centered care can have on encouraging stroke survivors' engagement in rehabilitation, increasing understanding, as well as supporting individual's emotional and psychological adjustment.

However, this was not an experience that was universally shared between the participants. Ian experienced interactions with HCPs that focused predominantly on diagnosis, there was no acknowledgment of Ian's personal needs or concerns.

Stroke had blind-sided me…my independence and resilience was being challenged. There was little talk, as far as I can recall, about recovery. I felt lost, vulnerable, guilty due to how little I had been impacted compared with others on the ward. (Ian, aged 53)

During his stay in hospital, Ian's psychological and emotional wellbeing was being impacted, as he describes feeling vulnerable and lost as a result of stroke. This indicates an absence of support and that he was feeling alone in his experience, as he feels different to the other survivors on the ward. Following a second stroke, Ian experienced this again as “all of the focus was on the diagnosis rather than the recovery.” In Ian's experience, there was little attention paid to his personal needs in the short-term or in relation to his long-term recovery.

Stroke survivors can find it frustrating when their input and experiences are not being listened to. When survivors do feel heard, and have a positive, supportive relationship with HCPs, it can have a profound effect on a survivor's recovery and their post-stroke outlook. As a result, participants highlighted the importance of HCPs acknowledging each survivor's subjective concerns and needs. Time should be taken to discuss these issues with stroke survivors and their families. Moreover, the input, opinions and experiences of stroke survivors and their families need to be acknowledged and listened to by HCPs, including decisions that relate to treatment. By doing this, stroke survivors will feel more informed about their condition and more in control of their care/treatment. Acknowledging the individual, and what their pre-stroke life was like, can help HCPs understand how stroke may impact an individual and potentially prepare them more adequately for discharge and life after stroke. These issues are discussed in greater detail in Lessons 5 and 6.

#### Lesson 5: Stroke Survivors Can Often Feel Unprepared for the Reality of Life After Stroke

The participants expressed feeling unprepared for the reality of leaving hospital and adjusting to the impact of stroke on their lives. Participants highlighted a lack of discussion with HCPs about what to expect when returning to life following stroke and the specific impact it may have on their subjective lives.

In Emma's case, she describes feeling institutionalized while at hospital, as she states “being in hospital, you're surrounded by people who are sick, it's expected.” However, following discharge, Emma recounts experiencing “embarrassment,” “a lack of independence,” and “people looking at [her] in [her] wheelchair.” Emma felt unprepared not only for the reality of how her impairments would restrict her capacity but also for the social difficulties she would encounter due to acquired disabilities. Multiple participants, including Emma, mention their initial expectation was that they would return to their normal, pre-stroke life. However, due to the impact of stroke, many struggle to return to pre-stroke roles, responsibilities or hobbies. This often-drastic change to a stroke survivor's sense of normality can be compounded as a survivor can face difficulties when interacting with the outside world due to their acquired disabilities. Alan highlighted the difficulties one can face adapting to life after stroke:

Getting used to being back at home was very tiring…I think that was due to the new challenges and the frustrations it brought…I find tasks like shopping difficult as I cannot reach certain items. I came across this often and quickly saw the world from a new angle, the country where I lived was not disabled friendly. (Alan, aged 59)

The participants' stories show the shock stroke survivors can feel returning home after stroke. The participants expressed that, prior to discharge, HCPs should help prepare stroke survivors for the potential impact stroke will have on an individual's life; this is something Emma addressed directly in her story:

I feel like work needs to be done to research other stroke survivor's experience of discharge so that conversations can be had with survivors facing discharge to prepare them. They may not be able to return to the job that they were doing, or return to previous hobbies that they had and this needs to be acknowledged. (Emma, aged 33)

While Emma presents the need to prepare stroke survivors for the potential reality of life after stroke, Alan presents a case in which HCPs provide a cautious and deterministic prediction in regards to recovery expectations, as he states: “My family was told that I would plateau at 6 months post-stroke and wherever I was at that point of my recovery is where I would stay.” When HCPs offer cautious recovery expectations such as this, there is potential to restrict stroke survivors' hopes for improvement and not accurately reflect their capacity to recover further. As a result, HCPs must strike a balance when discussing recovery expectations to ensure that survivors are aware of how stroke may substantially impact their daily lives, while also encouraging engagement in rehabilitation and belief that improvement is still possible.

However, coming to terms with the possible substantial impact stroke may have on a survivor's life is often a long-term and complex psychological process, that has the potential to challenge an individual indefinitely. In his story, William discusses the enduring psychological challenge he has faced post-stroke:

The medical team and therapists warned me that the recovery process would be fast for a week or two and would then flatten. I could not understand what this really meant. And still, almost 4 years later, cannot come to terms with [the impact of stroke]. (William, aged 63)

While it is important that the process of supporting stroke survivors come to terms with the impact of stroke must begin in a primary healthcare setting, experiences such as William's highlight the need for HCPs to also be aware of the often long-term nature of this process.

Conversations should be held with stroke survivors prior to their discharge from a primary healthcare setting, so that their expectations can be managed about what to expect when attempting to adjust to life after stroke. It is important for HCPs to strike a balance between being realistic about the potential extent of the impact a stroke may have on a survivor's life; while also not removing hope for the potential of continued long-term improvement. These conversations should take into account the possible impact of stroke on the subjective individual lives of stroke survivors. There needs to be acknowledgment of the long-term nature of stroke recovery and adaption to life after stroke. However, survivors may not be prepared to fully acknowledge this during their acute recovery, so it is important that survivors are directed toward possible routes of long-term support, an issue further expanded upon in Lesson 6.

#### Lesson 6: Adapting to Life After Stroke Is a Long-Term Process Requiring Long-Term Support

Study participants highlighted that adapting to life after stroke is a long-term process and that most stroke survivors have to contend with long-term disability and the enduring impact this may have on their individual and social lives. Emma describes the nature of this process in her story:

Almost 6 years after my stroke and I'm still adjusting. For me, I feel like it's a continuous process. Ongoing rehabilitation means that you need to change. And although I'm back doing things I enjoy, it's different because I'm different. It's been a huge adjustment psychologically, socially and physically. Long-terms effects of the stroke, such as fatigue, mean that I need to manage my time. It's been a challenging road to navigate and I'm still learning. (Emma, aged 33)

Coming to terms with and adapting to acquired disabilities is often a long-term and potentially indefinite process. The holistic impact of stroke can fundamentally change the way a survivor manages their life, and even if one has the capacity to re-engage with pre-stroke activities, it will likely not replicate pre-stroke engagement. However, in many cases, returning to pre-stroke activities and roles is no longer possible; an experience that was highlighted in Jessica's story:

I have lost friends as I could no longer go do the things that we used to do. I had to close my business and I missed out on a big part of my youth. I've missed out on so much, just doing [my daughter's] hair pretty, walking her to school […] Now I'm always a spectator in a lot that she does but I choose to see the silver lining that I'm still a part of her life. (Jessica, aged 33)

As evident, out with the complex process of coming to terms with dramatic changes in a survivor's personal life, survivors must also contend with changes in the way they interact with society, and how society interacts with them. In Alan's story, he illustrates the difficulties he faced as he began to interact with a world that was not “disabled friendly.” This can extend to an individual's search for work, as was Ian's experience:

My minor speech issues had impacted my search for work. Many interviews but not until one with the stroke charity was it not a factor. (Ian, aged 59)

Ian's experience reflects how stroke survivors can be faced with barriers preventing them from reintegrating into society that are out of their control. Helping stroke survivors break down these barriers is significant, as successfully re-engaging with society and/or (re-)establishing new social roles and responsibilities can bring stroke survivors value. Ian reflects the importance of this in his story, as he describes recovering “a sense of purpose and structure” after he began volunteering at a national stroke charity.

Successfully supporting survivors to re-engage in social roles and activities may help increase their ability to adjust positively to life following stroke. However, stroke survivors often report a lack of long-term support following discharge (19), and this was reflected in the participants' stories. In the cases where support post-stroke was discussed within the digital stories, the effects were often positive; such as Alan, who found that “speaking to a stroke psychologist helped [him] understand the challenges [he] faced and helped [him] with strategies to improve.” It is not the responsibility of HCPs based in the acute setting to deliver long-term support following stroke. However, better communication between acute and community services can play a key role in helping stroke survivors access support that can help them adjust (64).

As has been evidenced, it is often only after stroke survivors leave a primary healthcare setting that they begin to realize the extent to which stroke has impacted their lives. Many survivors face a variety of long-term challenges, often of a psychological and social nature, which have the capacity to endure indefinitely. As a result, it is important for HCPs to understand the subjective circumstances a stroke survivor may face following discharge. This knowledge can help HCPs to effectively direct stroke survivors toward beneficial post-stroke support that can help survivors and their families manage the initial transition following discharge, as well as support for their long-term adjustment to life following stroke, that is relevant to their individual set of circumstances. HCPs can further support the transition by increasing communication and co-operation with community-based stroke support teams and organizations.

## Discussion

This study has attempted to design a novel co-creation method through the integration of a co-production design framework with the BCW steps to co-create digital stories and apply it to identify core collective lessons through the synthesis of individual stroke survivors' experiences of interacting with healthcare professionals. Studies have shown the benefits of including stroke survivors and caregivers within the design process in order to develop more relevant and effective outcomes that emanate directly from the experiences of those who the intervention concerns most (65–68). The design of this study has placed the input of stroke survivors at the center of the co-creation method, which has subsequently resulted in the synthesis of six important collective lessons that help to detail the presence of preconceptions and attitudes embedded within healthcare that may be negatively impacting survivors' healthcare experiences, and the need for survivors' individual lived experiences to be acknowledged to achieve a truly person-centered approach throughout the stroke pathway.

### Implications for HCPs Working With Stroke Survivors

#### Misdiagnosis and Misconceptions

A central finding from this study was stroke survivors' experiences of misdiagnosis. This has pressing implications for healthcare services, as misdiagnosing “acute ischemic stroke is associated with adverse clinical outcomes including increased risk of stroke recurrence and increased mortality rates compared to stroke patients who are accurately diagnosed” (69) (p. 1). The participants of this study highlighted the significant role they believe assumptions in HCPs psychological capability play in driving misdiagnosis. These erroneous assumptions were in relation to the symptoms, age, and lifestyle choices typically believed to be associated with stroke.

The participants explained their belief that not displaying typical FAST symptoms contributed to their misdiagnosis. This is reflected in a study comparing 156 consecutive stroke patients misdiagnosed in an emergency department in Sydney, Australia, which found that patients who experienced stroke misdiagnosis were commonly FAST-negative and displayed non-specific or atypical symptoms (70). The problem of stroke misdiagnosis has seen calls for a broadening of the symptoms being considered during the initial stroke diagnosis period and improvements to the diagnosis process itself (69, 71). Studies into the adoption of utilizing BE-FAST (Balance, Eyes, Face, Arms, Speech, and Time) as a wider screening technique in acute intervention found that this helped reduce the amount of missed strokes (72, 73). However, it is important to acknowledge that stroke misdiagnosis is a multifaceted problem with various contributing factors; these extend not just to procedural measures but embedded bias within healthcare provision.

As was reflected in the participants' stories, one factor that contributes to misdiagnosis is often the age of the person being diagnosed. Newman-Toker et al. (74) found that adult patients under the age of 44 were seven times more likely to be misdiagnosed following stroke when compared to patients over the age of 75. Bhat et al. (75) have highlighted the role of “implicit bias” within clinicians in contributing to stroke misdiagnosis in younger survivors, believed to be a result of the way clinicians are taught to recognize stroke through identifying classical symptoms, signs and patient risk profiles. This can reinforce the idea of a “stereotypic presentation” of stroke through pattern recognition; resulting in those who do not meet these criteria being at increased risk of misdiagnosis (75) (p. 30). This profiling extends beyond age and can include a variety of criteria such as gender, typical stroke symptoms or traditional risk factors. This bias is not exclusive to the diagnosis period and can be embedded into the care environment that stroke survivors experience. As a result, younger stroke survivors can experience care that may not meet their specific needs and, following discharge, fall through the gaps of existing stroke services (61).

As stroke incidence rates increase in younger people (2) and non-individual risk factors, such as air pollution, continue to contribute further to the rise in stroke incidence (76), challenging this inherent bias within healthcare is of increased importance. The digital stories created provide HCPs with first-hand accounts of how assumptions relating to diagnosis and beyond have impacted stroke survivors' adjustment following stroke. These collective lessons can help target behaviors that inform misconceptions, which in turn lead to stroke misdiagnosis and the perpetuation of care focused on a “stereotypic presentation” of stroke.

#### Epistemic Injustice and Humility

The presence of implicit bias and the stereotypic presentation of stoke can be seen as a product of the endemic power imbalance between patients and healthcare providers often present within medical institutions. At the core of this imbalance is the HCPs role as a “gatekeeper” to knowledge and solutions to the medical condition a patient may have (77) (p. 206). This imbalance can be exacerbated by the pervasive prioritization of certain methods, practices, forms of evidence, and ways of knowing within contemporary healthcare (78) (p. 1,109). This divide can culminate in what Miranda Fricker (79) describes as *epistemic injustice*, which describes how knowledge claims can be unfairly dismissed, limiting an actor's epistemic credibility and capacity. In a healthcare setting, epistemic injustice occurs when patients are unfairly discredited by HCPs as being unreliable in their ability to provide information or knowledge on their own illness experiences (80, 81).

In this study, experiences of epistemic injustice were evidenced by the participants in their digital stories, as they reported having their concerns or input about their condition being downplayed or dismissed by HCPs; or through experiencing limited communication with HCPs about the concerns and issues that mattered to them most. Examples of epistemic injustice have been highlighted in prior stroke-related studies, such as: ineffective communication (82) (p. 19); inconsistent levels of HCPs stroke-related knowledge leading to reduced empathy (17); or difficulties communicating with survivors who have communicative impairments leading to survivors being less able to express their wishes and needs, and being excluded from care-related conversations (83).

To help challenge the issues that arise due to epistemic injustice, the culture that is embedded within healthcare institutions must be addressed. The concept of *epistemic humility* can help support change, as restricting one's confidence purely to the boundaries of their expertise and accepting one's own potential fallibility is encouraged (84). While also recognizing “that knowledge creation is an interdependent and collaborative activity” (84) (p. 117), and therefore, to fully understand a patient's experience, HCPs must work with patients to investigate any given healthcare issue. The effect of such an approach was noted by the participants, as they reported the positive impact more collaborative and inclusive interactions with HCPs can have on survivors feeling listened to and encouraged in their recovery. The importance of this approach has been reflected elsewhere as it can increase stroke survivors' confidence in the professionals' ability to take care of them (85); and increase survivors' involvement, motivation and coping (86). As a result, encouraging epistemic humility in HCPs could help to better support survivors throughout their interactions with HCPs from initial admission to life after stroke. Furthermore, it is important to acknowledge that, due to the inherent attitudes embedded within healthcare institutions, incidences of epistemic injustice are likely to manifest in the experiences of other patient groups.

#### Person-Centered Care

The digital stories demonstrated the significance of taking into account the subjective circumstances of individual stroke survivors throughout their stroke rehabilitation and adaption to life after stroke. Central to this was the quality of communication survivors received during their acute hospitalization, how unprepared they felt for discharge, and the long-term difficulties a survivor can face adapting to stroke.

Participants portrayed the importance of inclusive communication between stroke survivors and the HCPs treating them, where stroke survivors feel their individual needs are considered. This has been reflected elsewhere, as stroke survivors who report a perceived involvement in their care and treatment are associated with reporting their recovery service needs being met (87). More specifically, met needs have also been associated with shared decision-making in the rehabilitation goal-setting process (88, 89). Yet, when it comes to managing conversations about post-stroke recovery and adjustment with stroke survivors, the participants described how HCPs must find a balance between providing encouragement to engage with rehabilitation while managing expectations about the reality of potential long-term impairments. Healthcare staff have reported conservatively managing expectations in regards to recovery to avoid disappointment in stroke survivors if goals cannot be met (90). However, Scobbie et al. (91) suggest that rehabilitation staff should support the personal goals set by stroke survivors but to prepare for and anticipate potential failure in meeting their personal goals. By doing so, healthcare can begin to frame long-term adjustment post-stroke as a fluent and changing pathway, where stroke survivors' recovery aims, and their understanding of the way their acquired disabilities have impacted their lives, adapts over time.

Framing recovery expectations this way may be important as the digital stories highlighted how stroke survivors are often initially unprepared and unaware of the extent to which stroke will impact their lives. The shock of returning home (13–15) and the difficulties stroke survivors face as they navigate new challenges and experiences in relation to autonomy, uncertainty, engagement, hope and social relations (92) are well-documented. Participants highlighted the need for better preparation for the extent of these potential challenges prior to discharge. However, it is important to acknowledge that stroke survivors may not be prepared to understand the full extent of their newly changed lives in an acute setting, as stroke survivors have yet to face the potential fundamental change stroke can cause in regards to the discontinuity of their pre-stroke roles and sense of self (93). The participants highlighted how it is often only once stroke survivors leave the primary care setting that the reality of the subjective impact of stroke on their individual and social lives becomes apparent. As a result, establishing continuity of care and support is important to best support stroke survivors as they come to terms with the impact of stroke on their lives and the long-term challenges they may face (19). HCPs can play a key role in helping individuals prepare for their return home, as effective discharge communication is essential in establishing continuity of care and improving the level of support following discharge after stroke (64, 94, 95).

It is not only important for care to continue, as participants explained their desire for this care to be based around their individual needs and circumstance. The concept of person-centered care has been increasingly adopted across healthcare as an attempt to focus the delivery of care around the needs of the individual in question. This extends to stroke-related services, as person-centered care and rehabilitation frameworks have been increasingly developed and implemented (21–25). Yet, in the UK, gaps in the wholesale integration of this approach are still evident, as care deficiencies such as: abrupt discharge, poor communication and poor follow-up endure (96). This is characterized by the continuation of inadequate communication between healthcare sectors resulting in fragmented care and unmet stroke survivor needs (97). Many of these issues were reflected in the digital stories where the participants detailed the absence of patient-centered approaches during acute hospitalization, their return home, and long-term adjustment. As a result, in order to support the enaction of an effective person-centered approach, the concept of epistemic humility must be placed at the core of interactions between healthcare professionals and stroke survivors. It is only through the full acknowledgment of an individual's lived experience, and the subjective knowledge and insight they provide, that a truly person-centered approach may be implemented.

Therefore, to improve stroke survivors experience from acute care to discharge and long-term adjustment, establishing a continuity of care focused around the specific needs of the individual is essential. Central to the concept of person-centered care is the idea of active listening and self-awareness (98); which reflects the importance of HCPs embracing epistemic humility and inclusive, collaborative communication when working with stroke survivors. HCPs can play an important role in building a person-centered approach within acute settings by placing stroke survivors' input and subjective experiences at the center of the care they receive, while also helping to improve communication with post-discharge services in order to support the transfer of knowledge and care between settings. In doing so, HCPs can help contribute to a person-centered continuity of care from initial admission to life following discharge that is truly based around the individual.

### Co-creating Digital Stories With the BCW

This study reports on the ability to develop a behavior change intervention through the co-creation of digital stories achieved within a method that combines co-creation and the BCW. In a systematic review looking into digital storytelling in health professions education, Moreau et al. (44) found that patient digital stories, without input from HCPs, had minimal impact on health professionals' learning. However, we believe that by integrating the BCW within the co-creation process has resulted in the development of focused digital stories. These powerful stories have the potential to change HCPs' knowledge and attitudes and may ultimately change their behaviors toward stroke survivors as well. The BCW was found to provide the co-creation process with structure and purpose, while not compromising the process being led by the input and designs of the co-participants. As a result, the digital stories developed have resulted in a specific behavioral change intervention that emanates directly from the input and lived experiences of stroke survivors. Furthermore, this approach can be used in various settings, especially in regards to understanding healthcare experiences of survivors of other serious illnesses.

### Limitations

There should always be an application of caution when considering the findings of a study with a small number of participants, as it cannot be known whether the digital story content and the targeted behaviors would be replicated by other groups of stroke survivors. Furthermore, the co-creation process was also impacted by the necessary withdrawal of a participant toward the end of the design cycle. This meant this individual contributed toward stages 1, 2, and 3 of the co-creation process but did not create a digital story. As a result, they helped contribute to the content and design of the digital stories, however, their digital story was not present to support the important lessons the digital stories aim to address.

Efforts were made to ensure a variety of voices were included within the co-creation process through the implementation of a maximum variation sample. However, while there is a spread of gender and socio-economic status, certain groups were not represented by this sample. Firstly, measures were put in place to enable the participation of stroke survivors who are unable to verbally communicate their experiences, however none such participants took part. As this study follows-up a previous qualitative study, it was already known that the subset of participants taking part would not have such impairments. This meant that an important demographic of stroke survivors was not represented in this study. Future studies should consider the integration of different forms of communication, such as the Talking Mats that were available for this study, to ensure these important voices and experiences are included. Furthermore, an age range spanning from 33 to 63 excludes voices of stroke survivors from several age groups, and specifically has resulted in more elderly participants not contributing. It is also important to note that the two youngest participants of this study who completed a digital story (both aged 33) were women; moreover, the three remaining participants to complete a digital story were all male, aged, 53, 59, and 63. This means a male perspective under the age of 53 and a female perspective over the age of 33 were not present in the sample. Furthermore, the sample of this study consists entirely of White British individuals, meaning voices of other ethnicities living with stroke in the UK were not represented in this study. Moreover, none of the participants contributing to this project had a disability prior to their stroke. As a result, the absence of such groups from the sample will likely have influenced the nature of the key lessons that were identified. This has the potential to limit the dependability and transferability (58) of the study findings, as stroke survivors from different demographical groups and backgrounds will likely have varied experiences in regards to their interactions with HCPs.

However, the purpose of this study was not to engage with a representative sample of stroke survivors. This study showed that strong consistent themes can emerge between individual stroke survivor stories. As a result, digital storytelling can be successfully utilized as a method to gather and synthesize important collective lessons about experiences post-stroke that can be used as an intervention technique to educate those interacting with people after stroke.

### Implications for Future Research

After the development of the digital stories, the next step is to engage HCPs with the intervention strategy and evaluate its impact on HCPs' psychological capability. Looking forward, co-creating digital stories with support of the BCW could be further utilized as a tool to engage other stroke-related stakeholders to inform and encourage targeted behavior change. Moreover, this approach can be applied to help explore healthcare experiences of survivors of other serious illnesses. Further work is also required to better understand the impact of embedded cultural attitudes and behaviors within healthcare, not only the experiences of stroke survivors, but patient groups across the medical context.

## Conclusion

Co-creating digital stories through the integration of the Double Diamond design process and the BCW has enabled the production of an intervention strategy for HCPs working with people after stroke. Through this novel method, this study aimed to identify core collective lessons for healthcare professionals working with people after stroke through the synthesis of stroke survivors experience of healthcare. We believe that the findings of this study support the need for a truly person-centered approach to be implemented throughout the stroke recovery pathway. This includes HCPs adopting the concept of epistemic humility so that all interactions with stroke survivors, from initial hospitalization to life after stroke, place their lived experience and input at the core of the care they receive. In order to do so at a patient level, HCPs must be receptive to all relevant person-specific information and this must be communicated effectively between the various support networks survivors engage with throughout their post-stroke journey. Furthermore, in order to do this at an organizational level, it is important for HCPs to understand and respond to the institutionalized behavior that is formed through the hierarchical structure inherent in healthcare. We believe that if these behaviors are changed to help address implicit bias and epistemic injustice within healthcare, it may help to increase the value of patient voices; reduce misdiagnosis; improve healthcare experiences and outcomes for stroke survivors; and lead to a more positive adjustment to life after stroke.

## Data Availability Statement

The raw data supporting the conclusions of this article will be made available by the authors, without undue reservation.

## Ethics Statement

The studies involving human participants were reviewed and approved by Glasgow Caledonian University's Glasgow School for Business and Society Research Ethics Committee and the University Ethics Committee. The patients/participants provided their written informed consent to participate in this study.

## Author Contributions

JH was the primary researcher conducting the research and analysis. HB-M, FW, and TK supervised the research process as director of studies and co-supervisors. All authors contributed to the article and approved the submitted version.

## Funding

This study was funded as a part of a full-time Ph.D. research studentship at Glasgow Caledonian University within the Glasgow School for Business and Society (Proj. Ref No. GSBS2018004BassilM).

## Conflict of Interest

The authors declare that the research was conducted in the absence of any commercial or financial relationships that could be construed as a potential conflict of interest.

## Publisher's Note

All claims expressed in this article are solely those of the authors and do not necessarily represent those of their affiliated organizations, or those of the publisher, the editors and the reviewers. Any product that may be evaluated in this article, or claim that may be made by its manufacturer, is not guaranteed or endorsed by the publisher.

## References

[1] Feigin VL Stark BA Johnson CO Roth GA Bisignano C. Global, regional, and national burden of stroke and its risk factors, 1990–2019: a systematic analysis for the Global Burden of Disease Study 2019. Lancet Neurol. (2021) 20:795–820. 10.1016/S1474-4422(21)00252-0 34487721 PMC8443449

[2] Ekker MS Boot EM Singhal AB Tan KS Debette S Tuladhar AM. Epidemiology, aetiology, and management of ischaemic stroke in young adults. Lancet Neurol. (2018) 17:790–801. 10.1016/S1474-4422(18)30233-3 30129475

[3] Virani SS Alonso A Benjamin EJ Bittencourt MS Callaway CW Carson AP. Heart disease and stroke statistics-−2020 update: a report from the American Heart Association. Circulation. (2020) 141:e139–596. 10.1161/CIR.0000000000000746 31992061

[4] Wafa HA Wolfe CDA Emmet E Roth GA Johnson CO Wang Y. Burden of stroke in Europe. Thirty-year projections of incidence, prevalence, deaths, and disability-adjusted life years. Stroke. (2020) 51:2418–27. 10.1161/STROKEAHA.120.029606 32646325 PMC7382540

[5] Cramer SC Wolf SL Adams HP Chen D Dromerick AW Dunning K. Stroke recovery and rehabilitation research: issues, opportunities, and the National Institutes of Health Stroke Net. Stroke. (2017) 48:813–9. 10.1161/STROKEAHA.116.015501 28174324 PMC5330812

[6] Hewitt G Sims S Greenwood N Jones F Ross F Harris R. Interprofessional teamwork in stroke care: is it visible or important to patients and carers? J Interprof Care. (2015) 29:331–8. 10.3109/13561820.2014.950727 25158116

[7] Aadal L Angel S Langhorn L Pedersen BB Dreyer P. Nursing roles and functions addressing relatives during in-hospital rehabilitation following stroke. Care needs and involvement. Scand J Caring Sci. (2018) 32:871–9. 10.1111/scs.12518 28869654

[8] Theadom A Rutherford S Kent B McPherson K. The process of adjustment over time following stroke: a longitudinal qualitative study. Neuropsychol Rehabil. (2018) 29:1464–74. 10.1080/09602011.2018.1440609 29480073

[9] Hartford W Lear S Nimmon L. Stroke survivors' experiences of team support along their recovery continuum. BMC Health Serv Res. (2019) 19:723. 10.1186/s12913-019-4533-z 31638959 PMC6805495

[10] Lawrence L Kinn S. Defining and measuring patient-centered care: an example from a mixed-methods systematic review of the stroke literature. Health Expect. (2011) 15:295–326. 10.1111/j.1369-7625.2011.00683.x 21624025 PMC5060626

[11] Harrison M Ryan T Gardiner C Jones A. Psychological and emotional needs, assessment and support post-stroke: a multi-perspective qualitative study. Top Stroke Rehabil. (2017) 24:119–25. 10.1080/10749357.2016.1196908 27309492

[12] Pallesen H. Body, coping and self-identity. A qualitative 5-year follow-up study of stroke. Disabil Rehabil. (2014) 36:232–41. 10.3109/09638288.2013.788217 23631656

[13] Hodson T Aplin T Gustafsson L. Understanding the dimensions of home for people returning home post stroke rehabilitation. Br J Occup Ther. (2016) 79:427–33. 10.1177/0308022615619420

[14] Pringle J Drummond JS McLafferty E. Revisioning, reconnecting and revisiting: the psychosocial transition of returning home from hospital following a stroke. Disabil Rehabil. (2013) 35:1991–9. 10.3109/09638288.2013.770081 23614358

[15] Connolly T Mahoney E. Stroke survivors' experiences transitioning from hospital to home. J Clin Nurs. (2018) 27:3979–87. 10.1111/jocn.14563 29893039

[16] Souter C Kinnear A Kinnear M Mead G. Optimisation of secondary prevention of stroke: a qualitative study of stroke patients' beliefs, concerns and difficulties with their medicines. Int J Pharm Pract. (2014) 22:424–32. 10.1111/ijpp.12104 24606322

[17] Tholin H Forsberg A. Satisfaction with care and rehabilitation among people with stroke, from hospital to community care. Scand J Caring Sci. (2014) 28:822–9. 10.1111/scs.12116 24495250

[18] Martinsen R Kirkevold M Sveen U. Young and midlife stroke survivors' experiences with the health services and long-term follow-up needs. J Neurosci Nurs. (2015) 47:27–35. 10.1097/JNN.0000000000000107 25565592

[19] Pindus DM Mullis R Lim L Wellwood I Rundell AV Aziz NAA. Stroke survivors' and informal caregivers' experiences of primary care and community healthcare services – a systematic review and meta-ethnography. PLoS ONE. (2018) 13:e0192533. 10.1371/journal.pone.0192533 29466383 PMC5821463

[20] Tunney AM Ryan A. Listening to carers' views on stroke services. Nurs Older People. (2014) 26:28–31. 10.7748/nop2014.02.26.1.28.e500 24471551

[21] Bushnell CD Duncan PW Lycan SL Condon CN Pastva AM Lutz BJ. A person-centered approach to poststroke care: the comprehensive post-acute stroke services model. J Am Geriatr Soc. (2018) 66:1025–30. 10.1111/jgs.15322 29572814 PMC9257530

[22] Dacosta D Dodds LJ Corlett SA. Development of a tool to support person-centered medicine-focused consultations with stroke survivors. Patient Educ Counsel. (2019) 102:1263–72. 10.1016/j.pec.2019.02.007 30765119

[23] Donnellan C Martins A Conlon A Coughlan T O'Neill D Collins DR. Mapping patients' experiences after stroke onto a patient-focused intervention framework. Disabil Rehabil. (2013) 35:483–91. 10.3109/09638288.2012.702844 22889261

[24] Dörfler E Kulnik ST. Despite communication and cognitive impairment - person-centered goal-setting after stroke: a qualitative study. Disabil Rehabil. (2020) 42:3628–37. 10.1080/09638288.2019.1604821 31020863

[25] Kidd L Booth J Lawrence M Rowat A. Implementing supported self-management in community-based stroke care: a secondary analysis of nurses' perspectives. J Clin Med. (2020) 9:985. 10.3390/jcm9040985 32244792 PMC7230474

[26] Bersano A Kraemer M Touzé E Weber R Alamowitch S Sibon I. Stroke care during the COVID-19 pandemic: experience from three large European countries. Eur J Neurol. (2020) 27:1794–800. 10.1111/ene.14375 32492764 PMC7300856

[27] Markus HS Martins S. COVID-19 and stroke—understanding the relationship and adapting services. A global World Stroke Organisation perspective. Int J Stroke. (2021) 16:241–7. 10.1177/17474930211005373 33709834 PMC8044614

[28] Zafra-Tanaka JH Portocarrero A Abanto C Zunt JR Miranda JJ. Managing post-stroke care during the COVID-19 pandemic at a tertiary care level hospital in Peru. J Stroke Cerebrovasc Dis. (2021) 106275. 10.1016/j.jstrokecerebrovasdis.2021.106275 35121533 PMC8702405

[29] Greenhalgh T Hurwitz B. Narrative based medicine: why study narrative? BMJ. (1999) 318:48–50. 10.1136/bmj.318.7175.48 9872892 PMC1114541

[30] Alawafi R Rosewilliam S Soundy A. An integrative review considering the impact of storytelling and sharing interventions in stroke. Behav Sci. (2021) 11:88. 10.3390/bs11060088 34208441 PMC8234102

[31] Balakrishnan R Kaplan B Negron R Kezhan F Goldfinger JZ Horowitz CR. Life after stroke in an urban minority population: a photovoice project. Int J Environ Res Public Health. (2017) 14:293. 10.3390/ijerph14030293 28287467 PMC5369129

[32] Maratos M Huynh L Tan J Lui J Jarus T. Picture this: exploring the lived experience of high-functioning stroke survivors using photovoice. Qualit Health Res. (2016) 26:1055–66. 10.1177/1049732316648114 27194645

[33] Törnbom K Lundälv J Palstam A Sunnerhagen KS. ‘My life after stroke through a camera lens'- a photovoice study on participation in Sweden. PLoS ONE. (2019) 14:e0222099. 10.1371/journal.pone.0222099 31509564 PMC6738637

[34] Lambert J. Digital Storytelling: Capturing Lives, Creating Community. Berkeley, CA: Digital Diner (2018). 10.4324/9781351266369

[35] Funaki S Fujita S Taiencho K. Creating community engagements between people with disability and the local community through digital storytelling. IAFOR J Cultural Stud. (2016) 1:5. 10.22492/ijcs.1.1.05

[36] Lenette C Brough M Cox L. Everyday resilience: narratives of single refugee women with children. Qualit Soc Work. (2013) 12:637–53. 10.1177/1473325012449684

[37] Rice C Chandler E Harrison E Liddiard K Ferrari M. Project re-vision: disability at the edges of representation. Disabil Soc. (2015) 30:513–27. 10.1080/09687599.2015.1037950

[38] Gladwin D. Digital storytelling going viral: using narrative empathy to promote environmental action. Media Practice Educ. (2020) 21:275–88. 10.1080/25741136.2020.1832827

[39] Hardy P Sumner T. Cultivating compassion: how digital storytelling is transforming healthcare. 1st ed. Cham: Springer International Publishing (2018). Available online at: https://link-springer-com.gcu.idm.oclc.org/book/10.1007/978-3-319-64146-1

[40] Harland N. Increasing empathy: digital storytelling in professional development. In: Hardy P Sumner T, editors, Cultivating Compassion: How Digital Storytelling is Transforming Healthcare. Cham: Springer International Publishing (2018). p. 267–77. Available online at: https://link-springer-com.gcu.idm.oclc.org/book/10.1007/978-3-319-64146-1 (accessed July 20, 2021).

[41] Sljivic H Sutherland I Stannard C Ioppolo C Morrisby C. Changing attitudes towards older adults: eliciting empathy through digital storytelling. Gerontol Geriatr Educ. (2021) 2021:1900838. 10.1080/02701960.2021.1900838 35837695

[42] Lal S Donnelly C Shin J. Digital storytelling: an innovative tool for practice, education, and research. Occup Ther Health Care. (2015) 29:54–62. 10.3109/07380577.2014.958888 25338054

[43] Marín VI Tur G Challinor J. An interdisciplinary approach to the development of professional identity through digital storytelling in health and social care and teacher education. Soc Work Educ. (2018) 37:396–412. 10.1080/02615479.2017.1408790

[44] Moreau KA Eady K Sikora L Horsley T. Digital storytelling in health professions education: a systematic review. BMC Med Educ. (2018) 18:208–208. 10.1186/s12909-018-1320-1 30200945 PMC6131857

[45] Petty J. Using arts-based digital storytelling in neonatal care to enhance nursing students' empathy. Nurs Child Young People. (2021) 2021:e1351. 10.7748/ncyp.2021.e1351 33522722

[46] Frank AW. The Wounded Storyteller: Body, Illness, and Ethics. 2nd ed. Chicago, IL: The University of Chicago Press (2013).

[47] Different Strokes,. Stroke Survivor Stories. (2021). Available online at: https://differentstrokes.co.uk/what-we-do/survivors-stories/ (accessed November 15, 2021).

[48] Stroke Association. Stroke Survivor Stories. (2021). Available online at: https://www.stroke.org.uk/#123 (accessed November 15, 2021).

[49] Patient Voices. The Patient Voices digital stories. (2021) http://www.patientvoices.org.uk/stories-htm (accessed November 15, 2021).

[50] UK Design Council. What Is the Framework for Innovation? Design Council's Evolved Double Diamond. Design Council (2020). Available online at: https://www.designcouncil.org.uk/news-opinion/what-framework-innovation-design-councils-evolved-double-diamond (accessed October 21, 2021).

[51] Michie S Atkins L West R. The Behaviour Change Wheel: A Guide to Designing Interventions. Sutton: Silverback (2014).

[52] Pyykkö H Suoheimo M Walter S. Approaching sustainability transition in supply chains as a wicked problem: systematic literature review in light of the evolved double diamond design process model. Processes. (2021) 9:2135. 10.3390/pr9122135

[53] Michie S van Stralen MM West R. The behaviour change wheel: a new method for characterising and designing behaviour change interventions. Implement Sci. (2011) 6:42. 10.1186/1748-5908-6-42 21513547 PMC3096582

[54] Murphy J Cameron L. The effectiveness of Talking Mats with people with intellectual disability. Br J Learn Disabil. (2008) 36:232–41. 10.1111/j.1468-3156.2008.00490.x

[55] Scottish Government. Scottish Index of Multiple Deprivation. (2020). Available online at: https://www.gov.scot/collections/scottish-index-of-multiple-deprivation-2020/ (accessed October 15, 2021).

[56] UK Government Ministry Ministry of Housing Communities Local Government. English Indices of Deprivation. (2019). Available online at: https://www.gov.uk/government/statistics/english-indices-of-deprivation-2019 (accessed October 15, 2021).

[57] Braun V Clarke V. Thematic Analysis: A Practical Guide. London: SAGE Publications (2022).

[58] Lincoln YS Guba EG. Naturalistic Inquiry. Newbury Park, CA: Sage Publications (1985). 10.1016/0147-1767(85)90062-8

[59] Michie S Richardson M Johnston M Abraham C Francis J Hardeman W. The behavior change technique taxonomy (v1) of 93 hierarchically clustered techniques: building an international consensus for the reporting of behavior change interventions. Ann Behav Med. (2013) 46:81–95. 10.1007/s12160-013-9486-6 23512568

[60] National Health Service (NHS) England. Stroke Act FAST. (2021). Available online at: https://www.nhs.uk/actfast/Documents/Act-FAST-A5-leaflet-white-man.pdf (accessed November 15, 2021).

[61] Sadler E Daniel K Wolfe CDA McKevitt C. Navigating stroke care: the experiences of younger stroke survivors. Disabil Rehabil. (2014) 36:1911–7. 10.3109/09638288.2014.882416 24467678

[62] Goffman E. Stigma: Notes on the Management of Spoiled Identity. Middlesex: Penguin Publishing. (1990).

[63] Boehme AK Esenwa C Elkind MSV. Stroke risk factors, genetics, and prevention. Circ Res. (2017) 120:472–95. 10.1161/CIRCRESAHA.116.308398 28154098 PMC5321635

[64] Sheehan J Lannin NA Laver K Reeder S Bhopti A. Primary care practitioners' perspectives of discharge communication and continuity of care for stroke survivors in Australia: a qualitative descriptive study. Health Social Care Community. (2021) 5:13696. 10.1111/hsc.13696 34957626

[65] Kulnik ST Pöstges H Townsend R Micklethwaite P Jones F. A gift from experience: co-production and co-design in stroke and self-management. Design Health. (2019) 3:98–118. 10.1080/24735132.2019.1577524

[66] Krieger T Floren M Feron F Dorant E. Optimising a complex stroke caregiver support programme in practice: a participatory action research study. Educ Act Res. (2021) 29:37–59. 10.1080/09650792.2019.1699131

[67] Nasr N Leon B Mountain G Nijenhuis SM Prange G Sale P Amirabdollahian F. The experience of living with stroke and using technology: opportunities to engage and co-design with end users. Disabil Rehabil. (2016) 1:653–60. 10.3109/17483107.2015.1036469 25879304

[68] Stewart C Power E McCluskey A Kuys S Lovarini M. Evaluation of a staff behaviour change intervention to increase the use of ward-based practice books and active practice during inpatient stroke rehabilitation: a phase-1 pre–post observational study. Clin Rehabil. (2020) 34:607–16. 10.1177/0269215520911420 32204599

[69] Bakradze E Liberman AL. Diagnostic error in stroke—reasons and proposed solutions. Curr Atheroscler Rep. (2018) 20:1–13. 10.1007/s11883-018-0712-3 29441421

[70] Venkat A Cappelen-Smith C Askar S Thomas PR Bhaskar S Tam A. Factors associated with stroke misdiagnosis in the emergency department: a retrospective case-control study. Neuroepidemiology. (2018) 51:123–7. 10.1159/000491635 30092562

[71] Wallace EJ Liberman AL. Diagnostic challenges in outpatient stroke: stroke chameleons and atypical stroke syndromes. Neuropsychiatr Dis Treat. (2021) 17:1469–80. 10.2147/NDT.S275750 34017173 PMC8129915

[72] Aroor S Singh R Goldstein LB. BE-FAST (balance, eyes, face, arm, speech, time): reducing the proportion of strokes missed using the FAST mnemonic. Stroke. (2017) 48:479–81. 10.1161/STROKEAHA.116.015169 28082668

[73] El Ammar F Ardelt A Del Brutto VJ Loggini A Bulwa Z Martinez RC. BE-FAST: a sensitive screening tool to identify in-hospital acute ischemic stroke. J Stroke Cerebrovasc Dis. (2020) 29:104821. 10.1016/j.jstrokecerebrovasdis.2020.104821 32312632

[74] Newman-Toker DE Moy E Valente E Coffey R Hines AL. Missed diagnosis of stroke in the emergency department: a cross-sectional analysis of a large population based sample. Diagnosis. (2014) 1:155–66. 10.1515/dx-2013-0038 28344918 PMC5361750

[75] Bhat A Mahajan V Wolfe N. Implicit bias in stroke care: a recurring old problem in the rising incidence of young stroke. J Clin Neurosci. (2021) 85:27–35. 10.1016/j.jocn.2020.12.017 33581786

[76] Lee KK Miller MR Shah ASV. Air pollution and stroke. J Stroke. (2018) 20:2–11. 10.5853/jos.2017.02894 29402072 PMC5836577

[77] Chung R. Structural health vulnerability: health inequalities, structural and epistemic injustice. J Soc Philos. (2021) 52:201–16. 10.1111/josp.12393

[78] Paton M Naidu T Wyatt TR Oni O Lorello GR Najeeb U. Dismantling the master's house: new ways of knowing for equity and social justice in health professions education. Adv Health Sci Educ. (2020) 25:1107–26. 10.1007/s10459-020-10006-x 33136279 PMC7605342

[79] Fricker M. Epistemic Injustice Power and the Ethics of Knowing. Oxford: Oxford University Press (2007). 10.1093/acprof:oso/9780198237907.001.0001

[80] Heggen KM Berg H. Epistemic injustice in the age of evidence-based practice: the case of fibromyalgia. Human Soc Sci Commun. (2021) 8:1–6. 10.1057/s41599-021-00918-3

[81] Carel H Kidd IJ. Epistemic injustice in healthcare: a philosophial analysis. Med Health Care Philos. (2014) 17:529–40. 10.1007/s11019-014-9560-2 24740808

[82] Cecil R Parahoo K Thompson K McCaughan E Power M Campbell Y. The hard work starts now: a glimpse into the lives of carers of community-dwelling stroke survivors. J Clin Nurs. (2011) 20:1723–30. 10.1111/j.1365-2702.2010.03400.x 20815862

[83] Chu C Sorin-Peters R Sidani S De La Huerta B McGilton KS. An interprofessional communication training program to improve nurses' ability to communicate with stroke patients with communication disorders. Rehabil Nurs. (2018) 43:E25–34. 10.1097/rnj.0000000000000041 30395560

[84] Ho A. Trusting experts and epistemic humility in disability. Int J Feminist Approaches Bioethics. (2011) 4:102. 10.3138/ijfab.4.2.102

[85] Taule T Strand LI Skouen JS Råheim M. Striving for a life worth living: stroke survivors' experiences of home rehabilitation. Scand J Caring Sci. (2015) 29:651–61. 10.1111/scs.12193 25648326

[86] Solakken LM Nordhaug M Halvorsen K. Patients' experiences of involvement, motivation and coping with physiotherapists during subacute stroke rehabilitation – a qualitative study. Eur J Physiother. (2022) 2022:2032825. 10.1080/21679169.2022.2032825

[87] Ytterberg C Kristensen HK Tistad M von Koch L. Factors related to met needs for rehabilitation 6 years after stroke. PLoS ONE. (2020) 15:e0227867. 10.1371/journal.pone.0227867 31940423 PMC6961904

[88] Kristensen HK Tistad M von Koch L Ytterberg C. The importance of patient involvement in stroke rehabilitation. PLoS ONE. (2016) 11:e0157149. 10.1371/journal.pone.0157149 27285997 PMC4902299

[89] Levack WM Weatherall M Hay-Smith EJ Dean SG McPherson K Siegert RJ. Goal setting and strategies to enhance goal pursuit for adults with acquired disability participating in rehabilitation. Cochr Database Syst Rev. (2015) 2015:CD009727. 10.1002/14651858.CD009727.pub2 26189709 PMC8941379

[90] Plant SE Tyson SF Kirk S Parsons J. What are the barriers and facilitators to goal-setting during rehabilitation for stroke and other acquired brain injuries? A systematic review and meta-synthesis. Clin Rehabil. (2016) 30:921–30. 10.1177/0269215516655856 27496701 PMC4978164

[91] Scobbie L Brady MC Duncan EAS Wyke S. Goal attainment, adjustment and disengagement in the first year after stroke: a qualitative study. Neuropsychol Rehabil. (2021) 31:691–709. 10.1080/09602011.2020.1724803 32412863

[92] Lou S Carstensen K Jørgensen CR Nielsen CP. Stroke patients' and informal carers' experiences with life after stroke: an overview of qualitative systematic reviews. Disabil Rehabil. (2017) 39:301–13. 10.3109/09638288.2016.1140836 26882958

[93] Satink T Cup EH Ilott I Prins J De Swart BJ Njihuis-van der Sander MW. Patients' views on the impact of stroke on their roles and self: a thematic synthesis of qualitative studies. Archiv Phys Med Rehabil. (2013) 94:1171–83. 10.1016/j.apmr.2013.01.011 23337428

[94] Miller KK Lin SH Neville M. From hospital to home to participation: a position paper on transition planning poststroke. Arch Phys Med Rehabil. (2019) 100:1162–75. 10.1016/j.apmr.2018.10.017 30465739

[95] Reeves MJ Hughes AK Woodward AT Freddolino PP Coursris CK Swierenga SJ. Improving transitions in acute stroke patients discharged to home: the Michigan stroke transitions trial (MISTT) protocol. BMC Neurol. (2017) 17:1. 10.1186/s12883-017-0895-1 28623892 PMC5474297

[96] Gallacher KI May CR Langhorne P Mair FS. A conceptual model of treatment burden and patient capacity in stroke. BMC Fam Pract. (2018) 19:9–9. 10.1186/s12875-017-0691-4 29316892 PMC5759246

[97] Aquino MRJR Mullis R Moore C Kreit E Lim L McKevitt C. It's difficult, there's no formula: qualitative study of stroke related communication between primary and secondary healthcare professionals. Int J Integr Care. (2020) 20:11. 10.5334/ijic.5465 33250676 PMC7664307

[98] Haley B Heo S Wright P Barone C Rao Rettiganti M Anders M. Relationships among active listening, self-awareness, empathy, and patient-centered care in associate and baccalaureate degree nursing students. NursingPlus Open. (2017) 3:11–6. 10.1016/j.npls.2017.05.001

